# Challenges in Biometry and Intraocular Lens Power Calculations in Keratoconus: A Review

**DOI:** 10.3390/diagnostics15243121

**Published:** 2025-12-08

**Authors:** Mayank A. Nanavaty

**Affiliations:** 1 Sussex Eye Hospital, University Hospitals Sussex NHS Foundation Trust, Brighton BN2 5BF, UK; mayank.nanavaty@nhs.net; 2 Brighton & Sussex Medical School, University of Sussex, Falmer, Brighton BN1 9PX, UK

**Keywords:** keratoconus, biometry, IOL power calculation, cataract surgery, Barrett’sTrue-K, Kane formula, total keratometry

## Abstract

**Purpose:** The purpose of this work was to conduct a comprehensive literature review of the challenges encountered in ocular biometry and intraocular lens (IOL) power calculations in patients with keratoconus undergoing cataract surgery and to evaluate the performance of various biometric techniques and IOL power calculation formulas in this population. **Methods:** A comprehensive literature search was conducted in PubMed for studies published until October 2025. Keywords included “keratoconus”, “biometry”, “IOL power calculation”, “cataract surgery”, “keratometry”, and related terms. Studies evaluating the repeatability of biometric measurement, the accuracy of IOL formulas, and surgical outcomes in keratoconus patients were included. Study quality was assessed using standardized criteria, including study design, measurement standardization, and statistical appropriateness. **Results:** Twenty studies comprising 1596 eyes with keratoconus were analyzed. Biometric challenges include reduced keratometry repeatability (especially with K > 55 D), altered anterior-to-posterior corneal curvature ratios, anterior chamber depth, unreliable corneal power measurements, and tear film instability affecting measurement consistency. Keratoconus-specific formulas (Barrett’s True-K for keratoconus and Kane’s formula for keratoconus) demonstrated superior accuracy compared to standard formulas. The Barrett True-K formula with predicted posterior corneal astigmatism showed median absolute errors of 0.10–0.35 D across all severity stages, with 39–72% of eyes within ±0.50 D of target refraction. Traditional formulas (excluding SRK/T) produced hyperopic prediction errors that increased with disease severity. Swept-source optical coherence tomography biometry with total keratometry measurements improved prediction accuracy, particularly in severe keratoconus. **Conclusions:** IOL power calculation in keratoconus remains challenging due to multiple biometric measurement errors. Keratoconus-specific formulas significantly improve refractive outcomes compared to standard formulas. The use of total keratometry and swept-source OCT biometry, as well as the incorporation of posterior corneal power measurements, enhances accuracy. A multimodal approach combining advanced biometry devices with keratoconus-specific formulas is recommended for optimal outcomes.

## 1. Introduction

Keratoconus is a progressive, non-inflammatory corneal ectatic disorder characterized by stromal thinning and conical protrusion of the cornea, resulting in irregular astigmatism, myopia, and visual impairment. The global prevalence of keratoconus is approximately 1.38 per 1000 population, with significant geographic and ethnic variations [1,2]. While keratoconus typically manifests during adolescence or early adulthood, patients with this condition develop cataracts at an earlier age compared to the general population, often necessitating surgical intervention [3,4].

This review focuses specifically on the biometric and IOL calculation challenges in keratoconus cataract patients rather than providing a general overview of keratoconus disease. Given the increasing incidence of keratoconus in certain populations and improvements in disease stabilization through corneal collagen cross-linking, an expanding population of keratoconus patients now reaches cataract age, necessitating cataract surgery. This has created a focused clinical problem: accurate IOL power calculation in these complex eyes. Understanding the specific biometric pitfalls and optimized formula approaches is essential for surgeons managing this increasingly common scenario.

Cataract surgery in eyes with keratoconus presents unique challenges, particularly regarding accurate intraocular lens (IOL) power calculation. The fundamental assumption underlying most IOL power calculation formulas—that the anterior-to-posterior corneal curvature ratio remains constant—is violated in keratoconic eyes [5,6,7]. This disruption, combined with irregular corneal topography, altered effective lens position (ELP) estimation, and measurement variability, frequently results in unexpected hyperopic refractive outcomes, particularly as disease severity increases [7,8,9].

Historically, traditional IOL power calculation formulas have shown limited accuracy in keratoconus, with mean absolute prediction errors ranging from 0.56 D to over 2.0 D depending on disease severity [1,9]. In severe keratoconus (mean keratometry > 55 D), using actual keratometric values can lead to the selection of inappropriately low IOL powers, resulting in extreme postoperative hyperopia and potentially requiring IOL exchange [1,10]. To address these challenges, keratoconus-specific IOL power calculation formulas have been developed, including modifications of the Barrett True-K and Kane formulas [8,11,12].

Recent advances in biometry technology, particularly swept-source optical coherence tomography (SS-OCT) and total keratometry (TK) measurements, offer improved corneal power assessment by incorporating posterior corneal curvature [2,7,13]. However, the optimal combination of biometry devices and calculation formulas for different stages of keratoconus remains an area of active investigation.

While previous reviews have addressed keratoconus and IOL calculations, significant advances since 2023 warrant a comprehensive 2025 update:

1. Technological Advances: Swept-source OCT biometry with total keratometry capability has become more widely available. Recent studies [2,13] demonstrate substantially improved accuracy with these devices, providing data not previously comprehensively reviewed.
2. Formula Evolution: Barrett’s True-K for keratoconus has undergone refinements, and recent validations [5,14,15] provide dramatically more data than earlier reviews. The network meta-analysis by Tian et al. [12] provides the most current comparative evidence across 637 eyes.
3. Quantitative Outcome Data: This review provides detailed severity-stratified quantitative outcomes (median absolute errors and percentage within ±0.50 D and ±0.25 D), enabling evidence-based formula selection, whereas previous reviews were more qualitative.
4. Data for the Validation of the Kane Formula: Since the work of Singh et al. [11], multiple independent validations of the Kane keratoconus formula have emerged, revealing more nuanced performance patterns (including myopic overcorrection in severe cases) not previously characterized.
5. Total Keratometry Evidence: The expanded evidence on total keratometry performance [2,13] represents substantial new data, improving understanding of how posterior corneal measurements enhance accuracy.
6. Practical Clinical Framework: This review synthesizes evidence into actionable clinical recommendations with severity-stratified decision algorithms not present in previous reviews.

Thus, our 2025 review provides (1) updated evidence from the literature published within the period of 2023–2025; (2) quantitative severity-stratified performance data, enabling formula selection; (3) a synthesis of total keratometry evidence; (4) characterization of the Kane formula’s more variable performance; and (5) a practical clinical implementation framework based on current evidence.

This comprehensive literature review aims to (1) identify and characterize the specific biometric challenges in keratoconus that compromise the accuracy of IOL power calculation, (2) compare the performance of various biometry techniques and devices, (3) evaluate the accuracy of different IOL power calculation formulas stratified by keratoconus severity, and (4) provide evidence-based recommendations for clinical practice.

## 2. Methods

### 2.1. Literature Search Strategy

A comprehensive literature search was conducted in PubMed from inception through October 2025. The search strategy employed Medical Subject Heading (MeSH) terms and keywords including “keratoconus”, “keratoconic”, “corneal ectasia”, “IOL power calculation”, “intraocular lens”, “biometry”, “keratometry”, “cataract surgery”, “phacoemulsification”, “Barrett True-K”, “Kane formula”, “total keratometry”, “swept-source OCT”, “Pentacam”, and “IOLMaster”. Boolean operators (AND, OR) were used to combine search terms appropriately.

### 2.2. Inclusion and Exclusion Criteria

Inclusion criteria as listed as follows:

- Studies evaluating IOL power calculation accuracy in keratoconus patients undergoing cataract surgery;
- Studies assessing repeatability and reproducibility of biometric measurements in keratoconus;
- Comparative studies of different IOL power calculation formulas in keratoconus;
- Studies reporting quantitative refractive outcomes (mean absolute error, median absolute error, and percentage within ±0.50 D or ±1.00 D);
- Retrospective and prospective clinical studies, case series (≥10 eyes), and comprehensive reviews;
- Full-text articles in English.

Exclusion criteria included the following:

- Studies with fewer than 10 keratoconic eyes;
- Case reports;
- Studies evaluating only post-keratoplasty eyes without native keratoconus data;
- Studies not reporting quantitative biometry or refractive outcomes;
- Non-English-language publications;
- Animal and in vitro studies.

### 2.3. Data Extraction and Analysis

Extracted included the following: study design, sample size, patient demographics, keratoconus severity classification, biometry device used, IOL power calculation formulas evaluated, mean/median absolute prediction errors, percentage of eyes within ±0.50 D and ±1.00 D of target refraction, and complications. Keratoconus severity was classified according to the modified Amsler–Krumeich classification: mild (mean K ≤ 48 D), moderate (mean K 48–53 D), and severe (mean K > 53 D) [8,16].

### 2.4. Quality Assessment and Risk of Bias

Study quality was assessed using a combination of approaches:

1. Methodological quality was evaluated using criteria adapted from the Cochrane Risk of Bias Tool, focusing on the following:
  - Study design (prospective vs. retrospective, which, itself, constitutes bias risk;
  - Clear definition and clarity of inclusion/exclusion criteria;
  - Baseline characteristics and demographic documentation;
  - Standardization of biometric measurement protocols;
  - Masking of outcome assessors for refractive outcomes (where applicable).
2. Measurement Quality:
  - Repeatability and reproducibility of biometric measurements (reported or inferable);
  - Completeness of follow-up and use of validated measurement devices;
  - Documentation of measurement conditions (number of scans and exclusion criteria for poor quality);
  - Consistency of refractive outcome assessment timing.
3. Statistical Quality:
  - Appropriate outcome measures (absolute error and percentage within targets);
  - Completeness of follow-up and handling of missing data;
  - Appropriate statistical methods;
  - Subgroup analysis by severity when applicable and acknowledgment of potential biases.
4. Reporting Quality:
  - Acknowledgment of limitations;
  - Discussion of potential sources of bias;
  - Transparency regarding constant optimization of formulas;
  - Disclosure of conflicts of interest and funding sources.

## 3. Results

### 3.1. Study Characteristics

The comprehensive literature search identified 496 potentially relevant articles. After screening titles and abstracts, 64 articles underwent full-text review. The lists of included and excluded studies are shown in Table 1 and Table 2. Twenty studies meeting the inclusion criteria were included in this comprehensive review, comprising 1596 eyes with keratoconus undergoing cataract surgery or IOL power calculation assessment. Study designs included retrospective case series (*n* = 14), prospective studies (*n* = 3), multicenter studies (*n* = 2), and one network meta-analysis. Sample sizes ranged from 12 to 637 eyes. The mean age of patients across studies ranged from 43.7 to 68 years, reflecting the earlier onset of cataract formation in keratoconus populations.

### 3.2. Heterogeneity and Methodological Variation

Substantial heterogeneity across the 20 included studies limited direct meta-analysis and required careful interpretation of the findings. Heterogeneity arose from several sources:

1. Keratoconus Classification Systems:
  - Modified Amsler–Krumeich classification (K-based) used by some studies;
  - Direct K values (>55 D threshold) used by others;
  - Pentacam-based grading (indices like Kmax) used in additional studies;
  - This classification variability prevents precise severity stratification across studies.
2. Biometry Platforms:
  - Pentacam (Scheimpflug tomography);
  - IOLMaster 500 (partial coherence interferometry);
  - IOLMaster 700 (swept-source OCT with total keratometry);
  - Anterion and ARGOS (alternative swept-source platforms);
  - Contact ultrasound biometry;
  - Different devices demonstrate varying repeatability and measured values in keratoconus.
3. IOL Power Reporting Metrics:
  - Mean absolute error (MAE) vs. median absolute error (MedAE);
  - Different percentage-within-target thresholds (±0.25 D, ±0.50 D, and ±1.00 D);
  - Prediction error (signed) vs. absolute error;
  - Some studies reporting percentages differently prevents direct comparison.
4. IOL Formula Comparisons:
  - Different formula combinations tested across studies;
  - Inconsistent optimization of formula constants;
  - Some studies comparing only 3–4 formulas; others comparing >10;
  - Varying versions of formulas (Barrett’s True-K with predicted vs. measured PCA).
5. Study Design and Sample Characteristics:
  - Retrospective (*n* = 14) vs. prospective (*n* = 3) designs;
  - Variable follow-up timing (1 month to 6+ months);
  - Participant age ranges vary from mid-40s to late 60s;
  - Inclusion of eyes with prior corneal procedures (keratoplasty and ICRS) vs. native keratoconus only;
  - Some studies include all keratoconus severities; others specifically focus on severe disease.
6. Outcome Timing:
  - Refractive assessments at 1 month, 3 months, 6 months, or variable intervals;
  - Some studies perform cycloplegic refraction; others manifest refraction;
  - Timing of outcome assessment relative to refractive stabilization varied.

Due to this heterogeneity, meta-analytic pooling of effect estimates was not appropriate. Instead, we performed descriptive synthesis organized by severity stage, enabling comparison of formulas across standardized disease strata despite methodological variation.

### 3.3. Biometric Challenges in Keratoconus (Table 3)

#### 3.3.1. Keratometric Measurement Variability

Keratometry readings represent the cornerstone of IOL power calculation, yet their reliability is significantly compromised in keratoconus. Multiple studies have documented that keratometric repeatability decreases progressively with increasing disease severity [19,21,66].

**Table 3:** Biometry measurement challenges in keratoconus.

Parameter	Measurement Issue	Device/Modality	KC Severity Impact	Reference
Keratometry (K) Repeatability	Reduced repeatability with increasing KC severity	Pentacam, IOLMaster, and manual keratometry	Worsens significantly with K > 55 D	[19,21,66]
Axial Length (AL) Repeatability	Better repeatability than K readings	Optical biometry and ultrasound	Minimal impact by severity	[1,29,44]
Maximum K (Kmax) Repeatability	Least repeatable parameter (1.23 D)	Scheimpflug tomography	Range: 0.32–1.62 D by stage	[21]
Flat K (K1) Repeatability	Most repeatable parameter (0.51 D)	Pentacam HR	Range: 0.40–0.54 D by stage	[21,46]
Anterior Chamber Depth	Significantly deeper in KC eyes	Swept-source OCT and Scheimpflug tomography	Deeper by ~0.30–0.40 mm vs. normal	[22]
Posterior Corneal Power	Steeper than normal; underestimated by standard formulas	Scheimpflug tomography and SS-OCT	Greater discrepancy in advanced KC	[24,52,55]
Central Corneal Thickness	Thinner; affects total corneal power calculation	Scheimpflug tomography	Reduces with severity	[46,67]
Total Keratometry vs. Standard K	TK improves prediction in KC eyes	IOLMaster 700 (SS-OCT)	More beneficial in severe KC	[2,13,25]

Hashemi et al. evaluated five different keratometry devices (Pentacam^®^, Placido topographer, Orbscan^®^, IOLMaste^®^, and a manual keratometer) in 78 keratoconic eyes stratified by severity [19]. In mild keratoconus (K ≤ 55 D), the Pentacam demonstrated the best repeatability, with an intraclass correlation coefficient (ICC) of 0.974 for maximum K, followed by manual keratometry. However, in advanced keratoconus (K > 55 D), all devices showed markedly reduced repeatability, with ICCs ranging from 0.823 to 0.890, indicating substantial measurement error that could significantly affect IOL power selection.

The maximum keratometry value (Kmax) was identified as the least repeatable parameter, with a repeatability coefficient of 1.23 D in keratoconic eyes compared to 0.61 D in normal eyes. This repeatability deteriorated further with increasing severity, ranging from 0.71 D in stage 1 keratoconus to 1.74 D in stage 4. Conversely, the flat keratometry value (K1) demonstrated better repeatability, with coefficients of 0.51 D for Pentacam and 0.54 D for autokeratometry [21]. This repeatability deteriorated further with increasing severity, ranging from 0.71 D in stage 1 keratoconus to 1.74 D in stage 4 [46]. Conversely, the flat keratometry value (K1) demonstrated better repeatability, with coefficients of 0.51 D for Pentacam and 0.54 D for autokeratometry, [21] suggesting that K1 may serve as a more stable reference point for progression monitoring.

#### 3.3.2. Anterior and Posterior Corneal Curvature Relationship

A fundamental challenge in keratoconus IOL calculations stems from the disrupted relationship between anterior and posterior corneal curvatures. Standard IOL formulas assume a fixed ratio (typically 1.21:1) that enables estimation of total corneal power from anterior measurements alone [5,6,7]. In keratoconus, however, the posterior cornea steepens disproportionately, with the anterior-to-posterior ratio decreasing to 1.25–1.45 in keratoconic eyes compared to the expected ratio [52].

This altered relationship leads to systematic overestimation of total corneal power when using anterior measurements alone, consequently resulting in selection of lower-power IOLs and producing hyperopic refractive surprises [1,7,9]. Studies comparing simulated keratometry (Sim K) with total corneal refractive power (TCRP) in keratoconus demonstrated that Sim K overestimates TCRP by an increasing margin as disease severity progresses, with differences ranging from 0.5 D in mild cases to over 2.0 D in advanced keratoconus [7,20,55].

#### 3.3.3. Axial Length Measurement

Interestingly, axial length (AL) measurements demonstrate significantly better repeatability and reproducibility than keratometry in keratoconic eyes [1,29,44]. Yagci et al. found that the repeatability and reproducibility of AL measurements using optical biometry in keratoconic eyes were high and comparable to those in normal eyes, with ICCs exceeding 0.99 [45]. This finding is particularly important because errors in AL measurement contribute approximately 36% of total IOL power calculation error, compared to 22% from keratometry errors [13].

However, keratoconus eyes tend to have slightly longer axial lengths than age-matched normal eyes (mean, 24.39 mm; range, 21.82–28.69 mm), contributing to the myopic tendency often seen in these patients [29]. The relationship between AL and corneal power becomes particularly relevant for ELP estimation, as deeper anterior chambers (discussed below), combined with steep corneal measurements, may lead standard formulas to predict more posterior IOL positioning than actually occurs.

#### 3.3.4. Anterior Chamber Depth

Multiple studies have consistently shown that anterior chamber depth (ACD) is significantly deeper in keratoconic eyes than in normal eyes [32,56]. A systematic review and meta-analysis by Hashemi et al. found mean ACD differences of 0.30–0.40 mm in keratoconic eyes across multiple measurement devices [22]. This deepening correlates with the degree of corneal steepening and is thought to represent a compensatory mechanism or geometric consequence of the altered corneal curvature.

The clinical significance of deeper ACD lies in its impact on ELP prediction. Many IOL formulas use ACD as a variable for ELP estimation, but the algorithms were developed on normal eyes. In keratoconus, the relationship between preoperative ACD and postoperative IOL position may differ, potentially contributing to calculation errors. Interestingly, Wang et al. suggested that the measured ACD in keratoconus may be artificially magnified by the increased corneal curvature, meaning the “real” ACD could be shallower than measured [58]. This phenomenon adds another layer of complexity to IOL power calculation in these eyes.

#### 3.3.5. Central Corneal Thickness

Central corneal thickness (CCT) is characteristically reduced in keratoconus, with progressive thinning as disease severity increases. Mean CCT in keratoconus patients undergoing cataract surgery ranges from 445 to 555 μm, compared to approximately 540 μm in normal eyes [29,67].CCT becomes particularly important for newer IOL formulas that aim to calculate true total corneal power using thick-lens formulas that account for corneal thickness.

The Barrett True-K formula for keratoconus specifically incorporates CCT measurements to improve the estimation of total corneal power [12,15]. Studies have shown that incorporating CCT data improves formula accuracy, particularly in moderate and severe keratoconus, where posterior corneal contributions become more significant [24].

### 3.4. Device-Specific Considerations

*Scheimpflug tomography (Pentacam*) offers comprehensive anterior segment analysis, including both anterior and posterior corneal curvature measurements. In mild to moderate keratoconus (K ≤ 55 D), Pentacam demonstrates excellent repeatability for most parameters [19,46]. The device provides valuable indices that can improve IOL calculations, including true net power (TNP), total corneal power (TCP), and equivalent K readings (EKRs). However, repeatability decreases substantially in severe keratoconus [66].

*Swept-source OCT biometry devices (IOLMaster 700, Anterion, and ARGOS*) devices offer several advantages, including total keratometry measurements that incorporate posterior corneal power, higher success rates in dense cataracts, and improved resolution [25,33,43]. Comparative studies show swept-source OCT has better repeatability than Placido-Scheimpflug imaging for posterior corneal measurements in keratoconus [43]. The ability to measure total keratometry has been shown to improve IOL calculation accuracy in keratoconus, particularly when used with keratoconus-specific formulas [2,13,25].

*Partial Coherence Interferometry (IOLMaster 500):* While this device has excellent accuracy in normal eyes, it measures only anterior corneal curvature and has higher failure rates in dense cataracts. In keratoconus, the inability to assess posterior corneal power limits its utility for comprehensive IOL calculation [25,33].

*Ultrasound Biometry*: Contact ultrasound biometry has largely been superseded by optical methods. Studies comparing ultrasound versus optical biometry in post-penetrating keratoplasty eyes found no significant advantage of either method, with both failing to achieve target refraction in keratoconic eyes [16]. The contact nature and lower resolution make ultrasound less desirable for patients with keratoconus.

### 3.5. IOL Power Calculation Formulas: Performance Analysis (Table 4)

#### 3.5.1. Traditional Third-Generation Formulas

SRK/T: Among traditional formulas, SRK/T has consistently demonstrated the best performance in keratoconus eyes [1,9,17,68]. Multiple studies have ranked SRK/T as the most accurate third-generation formula, with mean absolute errors ranging from 0.56 D to 1.00 D depending on disease severity [8,15,17]. The superior performance of SRK/T in keratoconus appears to be related to its tendency toward myopic prediction error in eyes with steep corneal curvatures, which partially counterbalances the inherent hyperopic bias seen with other formulas [5,15].

**Table 4:** Performance comparison of IOL power calculation formulas in all keratoconus eyes.

Formula	Study	MAE (D)	MedAE (D)	Within ±0.50 D (%)	Within ±1.00 D (%)	Reference
Barrett’s True-K KC (P-PCA)	Vandevenne 2022	0.43 ± 0.42	0.14	72	NR	[15]
Barrett’s True-K KC (M-PCA)	Vandevenne 2022	0.44 ± 0.38	0.10	NR	90	[15]
Kane KC	Kane 2020	0.81	NR	NR	NR	[8]
SRK/T	Multiple	0.56–1.00	0.25–0.56	40–60	41–60	[1,3,9]
Barrett’s Universal II	Multiple	0.72 ± 0.58	0.47	NR	NR	[1,15]
EVO 2.0	Heath 2023	Variable	NR	NR	NR	[2]
Kane	Multiple	0.74 ± 0.63	0.50	NR	NR	[8,15]
Hoffer Q	Kane 2020	1.30	NR	NR	NR	[8]

MAE = mean absolute error; MedAE = median absolute error; NR = not reported; P-PCA = predicted posterior corneal astigmatism; M-PCA = measured posterior corneal astigmatism.

In mild keratoconus (K ≤ 48 D), Watson et al. reported that 60% of eyes achieved refractive outcomes within ±1.00 D of target when using actual K values with SRK/T [9]. However, in severe keratoconus (K > 55 D), the formula’s accuracy deteriorated substantially, with mean prediction errors exceeding 1.33 D [5].

Other third-generation formulas, including the Hoffer Q, Holladay 1, and Haigis formulas, generally perform poorly in keratoconus, with mean absolute errors of 1.18 D, 1.22 D, and 1.30 D, respectively, in Kane et al.’s series [8]. These formulas consistently produced hyperopic prediction errors that worsened with increasing corneal steepness, likely because their ELP prediction algorithms incorporate corneal power in ways that are inappropriate for keratoconic eyes.

#### 3.5.2. Fourth-Generation and Modern Formulae

Barrett’s Universal II (BUII), although not explicitly designed for keratoconus, has shown remarkable performance, particularly in mild-to-moderate cases. Helaly et al. found that BUII achieved a median absolute error of only 0.34 D across all severity stages, with 42.42% of eyes within ±0.25 D and 60.61% within ±0.50 D of target refraction [5]. In severe keratoconus, BUII maintained a MedAE of 0.46 D, which is comparable to the keratoconus-specific Barrett True-K formula (MedAE 0.56 D) [5]. This performance likely stems from BUII’s sophisticated ELP prediction algorithm, which incorporates axial length, anterior chamber depth, lens thickness, and white-to-white diameter, thereby reducing reliance on corneal power measurements [69].

The unmodified Kane formula shows moderate accuracy, with mean absolute errors of 0.74–1.05 D across studies [8,15]. Like other modern formulas, it tends to yield hyperopic prediction errors in keratoconus, with mean prediction errors of 0.60 D in Vandevenne et al.’s study and 1.74–2.04 D in severe cases [15].

EVO 2.0, a thick-lens formula based on Gaussian optics principles, considers anterior and posterior corneal curvatures and central corneal thickness [67]. Heath et al. found that when total keratometry was unavailable, EVO 2.0 K performed statistically better than Kane’s K in non-severe keratoconus [2]. Helaly et al. reported that EVO 2.0 achieved within ±1.00 D in 96.97% of eyes, the highest rate among the tested formulas, though its MedAE of 0.81 D was higher than that of Barrett-based formulas [5].

#### 3.5.3. Keratoconus-Specific Formulas

Barrett’s True-K for keratoconus is a formula that represents a modification of the post-refractive Barrett’s True-K, adapted specifically for keratoconus by incorporating measured or predicted posterior corneal power and central corneal thickness to estimate total corneal power [12,15]. The formula uses a “double-K” concept: one K value for ELP estimation and another for the vergence calculation to determine IOL power.

Performance data across multiple studies consistently demonstrate that Barrett’s True-K is one of the most accurate formulas for keratoconus:

- Vandevenne et al. (2022) [15]: In 57 eyes, Barrett’s True-K with predicted posterior corneal astigmatism (P-PCA) achieved a MedAE of 0.14 D, with 72% of eyes within ±0.50 D and 49% within ±0.25 D. The measured PCA version (M-PCA) achieved a MedAE of 0.10 D, with 90% within ±1.00 D [15]. Both versions significantly outperformed Barrett’s Universal II (MedAE of 0.47 D) and the Kane formula (MedAE of 0.50 D).
- Helaly et al. [5] (2025): In 99 eyes, including 33 with severe keratoconus, Barrett’s True-K KC achieved an overall MedAE of 0.35 D, with 39.39% within ±0.25 D [5]. Importantly, in severe cases (K > 53 D), the MedAE was 0.56 D, with 27.27% within ±0.25 D—substantially better than other formulas.
- Yokogawa et al. [14] (2024): In 131 Japanese eyes, Barrett’s True-K demonstrated the highest prediction accuracy among all tested formulas [14].
- Tian et al. [12] (2025): A Bayesian network meta-analysis of 637 eyes found Barrett’s True-K P-PCA and M-PCA to be ranked as the top two formulas for percentage of eyes within ±0.50 D, with statistically significant superiority over Barrett’s Universal II [12].

The formula’s performance remained relatively stable across severity stages, with MedAE increasing from 0.22–0.25 D in mild cases to 0.32–0.39 D in moderate and 0.56 D in severe keratoconus [5,15].

Introduced by Kane et al. [8] in 2020, Kane’s keratoconus formula modifies the original Kane formula by using a modified corneal power derived from the anterior corneal radius of curvature to compensate for the altered anterior/posterior ratio in keratoconus [8]. Additionally, it reduces the influence of corneal power on ELP prediction.

Kane et al.’s original study of 147 eyes found that their keratoconus formula achieved a mean absolute error of 0.81 D, significantly lower than all other tested formulas, including SRK/T (1.00 D), Barrett’s Universal II (1.03 D), and the unmodified Kane formula (1.05 D). Notably, 45% of eyes achieved refractive outcomes within ±0.50 D and 65% within ±1.00 D [8].

However, subsequent independent validations have yielded more variable results:

- Vandevenne et al. (2022) [15]: Kane’s KC showed a MedAE of 0.13 D overall, similar to Barrett’s True-K, but demonstrated a tendency toward myopic prediction error that increased with severity (mean PE −0.15 D in moderate KC and −0.75 D in severe KC) [15]. This myopic shift suggests the formula’s corneal power modification may overcorrect in more advanced cases.
- Helaly et al. [5] (2025): Kane’s KC was the only formula showing myopic mean prediction error (−0.76 ± 1.06 D) across all cases, with this myopic tendency becoming pronounced in severe cases (mean PE −1.50 ± 0.60 D and MedAE of 1.45 D) [5]. In mild keratoconus, the formula performed well (MedAE of 0.37 D), but accuracy deteriorated significantly with increasing severity.

The discrepancy between Kane’s original report and subsequent validations may relate to differences in constant optimization, biometry devices, or patient populations. The formula’s tendency toward increasing myopic error in more severe keratoconus suggests it may be overcorrecting for hyperopic bias and could benefit from severity-adjusted modifications.

Holladay 2 with Keratoconus Adjustment: This formula modifies the basic ELP algorithm to account for the anomalous relationship between axial length and anterior chamber depth in keratoconus [8]. However, Kane et al. [8] found it performed poorly, with a mean absolute error of 1.32 D, making it the least accurate formula in their series [8]. This formula has not been widely adopted or validated in subsequent studies.

#### 3.5.4. Severity-Stratified Analysis (Table 5)

##### Mild Keratoconus (Mean K ≤ 48 D)

In mild keratoconus, multiple formulas demonstrate acceptable performance. Most non-keratoconus-specific formulas achieve mean absolute errors under 0.50 D, with 40–60% of eyes within ±0.50 D of target [5,9,15]. Barrett’s Universal II performs exceptionally well in this subgroup, with Helaly et al. [5] reporting a MedAE of only 0.06 D and 54.55% of eyes within ±0.25 D [5].

**Table 5:** Formula performance stratified by keratoconus severity.

KC Severity	Formula	Study	MedAE (D)	Mean PE (D)	Within ±0.25 D (%)	Reference
Mild (≤48 D)	Barrett’s True-K KC	Vandevenne 2022	0.22–0.25	0.05 ± 0.59	36–55	[15]
Mild (≤48 D)	Barrett’s Universal II	Helaly 2025	0.06	0.12 ± 0.57	54.55	[5]
Mild (≤48 D)	SRK/T	Multiple	0.34	0.22 ± 0.54	NR	[1,9]
Mild (≤48 D)	Kane’s KC	Kane 2020	0.37	0.30 ± 0.66	54.55	[8]
Moderate (48–53 D)	Barrett’s True-K KC	Vandevenne 2022	0.32–0.39	0.28 ± 0.58	45.45	[15]
Moderate (48–53 D)	Barrett’s Universal II	Helaly 2025	0.33–0.34	0.17 ± 0.68	36.36	[5]
Moderate (48–53 D)	SRK/T	Vandevenne 2022	0.39	0.11 ± 0.70	NR	[15]
Moderate (48–53 D)	Kane’s KC	Vandevenne 2022	0.51–0.66	−1.05 ± 1.05	18.18	[15]
Severe (>53 D)	Barrett’s True-K KC	Helaly 2025	0.56	0.68 ± 0.52	27.27	[5]
Severe (>53 D)	Barrett’s Universal II	Helaly 2025	0.46	0.20 ± 0.66	27.27	[5]
Severe (>53 D)	SRK/T	Vandevenne 2022	1.33	0.72 ± 1.34	NR	[15]
Severe (>53 D)	Kane’s KC	Helaly 2025	1.45	−1.50 ± 0.60	0	[5]

PE = prediction error; negative values indicate myopic error; NR = not reported.

The original Kane and Kane KC formulas perform identically in mild cases when K ≤ 48 D, both achieving a MedAE of 0.37 D, with 54.55% within ±0.25 D [5]. This equivalence occurs because the keratoconus-specific modification does not significantly alter the calculation at lower corneal powers.

Even traditional formulas like SRK/T show reasonable accuracy in mild cases (MedAE of 0.34 D) [9,15]. This relatively good performance across multiple formulas suggests that the altered posterior corneal contribution in mild keratoconus is insufficient to compromise most modern IOL calculations substantially.

##### Moderate Keratoconus (Mean K 48–53 D)

Moderate keratoconus represents a transition zone where keratoconus-specific formulas begin demonstrating clear advantages. Barrett’s True-K maintains MedAE of 0.32–0.39 D, with 45.45% of eyes within ±0.25 D [15]. Barrett’s Universal II also performs well (MedAE of 0.33–0.34 D) but with a slightly lower percentage within strict tolerance (36.36% within ±0.25 D) [5].

Non-keratoconus formulas show deteriorating performance: The Kane formula’s MedAE increases to 0.56 D, with a mean prediction error of 0.51 D (hyperopic shift), EVO 2.0 shows a MedAE of 0.55 D, and Hoffer QST achieves a MedAE of 0.51 D [5].

A notable finding is that Kane’s KC begins showing myopic prediction error in moderate cases (mean PE −1.05 ± 1.05 D), with only 18.18% of eyes within ±0.25 D [5,15]. This suggests the formula’s corneal power modification may be excessive for this severity level.

##### Severe Keratoconus (Mean K > 53 D)

Severe keratoconus poses the greatest challenge for IOL power calculation, with accuracy deteriorating across all formulas. However, keratoconus-specific and advanced formulas maintain substantially better performance than traditional approaches.

Barrett’s True-K KC achieves a MedAE of 0.56 D, with 27.27% of eyes within ±0.25 D and 54.55% within ±1.00 D in severe cases. Remarkably, Barrett’s Universal II performs nearly as well (MedAE of 0.46 D, with 27.27% within ±0.25 D), suggesting its sophisticated ELP algorithm partially compensates for corneal power measurement errors, even without specific keratoconus adaptations [5].

In contrast, non-keratoconus-specific formulas show poor performance: SRK/T achieves a MedAE of 1.33 D with a large hyperopic shift (mean PE of 0.72 ± 1.34 D) [15], the Kane formula shows a MedAE of 1.90 D with severe hyperopic error (mean PE of 1.74–2.04 D [5,15], EVO 2.0 reaches a MedAE of 1.20 D, and Hoffer QST achieves a MedAE of 1.77 D [5].

Kane’s KC demonstrates concerning performance in severe cases, with a MedAE of 1.45 D and marked myopic prediction error (mean PE of −1.50 ± 0.60 D), resulting in 0% of eyes within ±0.25 D. ± 0.60 D [5]. This suggests that the formula’s modification substantially overcorrects in advanced disease.

Watson et al.’s earlier recommendation to use a standard K value of 43.25 D rather than actual measurements in severe keratoconus (K > 55 D) achieved a mean prediction error of +0.6 D, compared to +6.8 D when using actual K values, demonstrating the magnitude of hyperopic error produced by traditional approaches [9]. However, this strategy has largely been superseded by keratoconus-specific formulas that provide more accurate results while using actual measurements.

#### 3.5.5. Total Keratometry and Posterior Corneal Measurements

The introduction of total keratometry (TK) measurements by swept-source OCT biometers represents a significant advancement for keratoconus IOL calculations. TK directly measures both anterior and posterior corneal surfaces, eliminating the need to assume a fixed anterior-to-posterior ratio [2,7,13,25].

Heath et al. [2] (2023) specifically compared standard keratometry versus total keratometry in 87 keratoconic eyes [2]. Key findings included the following:

- Barrett’s True-K KC using TK with measured posterior corneal astigmatism (BU2 KCN:M-PCA) performed best across all severity subgroups, with significantly lower root mean square error than non-KC formulas.
- In severe KC, if TK values were unavailable, Barrett True-K KC with predicted PCA (BU2 KCN:P-PCA) performed better than the top-ranked non-KC formula (SRK/T).
- In non-severe KC without TK, EVO 2.0 K was statistically superior to Kane’s K.
- The use of TK consistently improved formula performance compared to standard K inputs.

Parra-Bernal et al. [13] (2024) evaluated 55 keratoconic eyes, comparing TK to standard keratometry across multiple formulas [13]. Barrett’s True-K KC with predicted PCA using standard keratometry registered the lowest mean absolute error and median absolute error. Importantly, when keratoconus-modified formulas were unavailable, entering TK into conventional formulas (rather than standard K) improved prediction outcomes, with all formulas showing a discrete increase in myopic error percentage with TK, suggesting TK produces more accurate corneal power estimates by accounting for posterior surface contributions [13].

These findings strongly support the use of swept-source OCT biometry devices capable of TK measurements in keratoconus patients, particularly when combined with formulas designed to utilize these posterior corneal data.

### 3.6. Special Considerations and Complicating Factors

#### 3.6.1. Ocular Surface Disease and Tear Film Instability

The increased prevalence of dry eye disease (DED) in keratoconus populations represents a significant but often overlooked factor affecting IOL power calculations and refractive outcomes. Multiple studies have documented increased tear osmolarity and dry-eye symptoms in keratoconus patients compared to age- and gender-matched control populations.

An unstable tear film compromises biometric measurements in several ways:

- Inconsistent keratometry readings due to irregular tear film and corneal surface irregularities;
- Altered anterior corneal power measurements with increased variability between sequential scans;
- Reduced reliability of optical biometry, necessitating multiple measurements;
- Potential degradation of corneal topography data quality.

Beyond measurement concerns, tear film instability impacts postoperative refractive outcomes through the following mechanism:

- Fluctuating visual acuity and refractive error during postoperative healing;
- Altered effective lens position calculation if pseudophakic ACD changes with tear film osmolarity;
- Reduced contrast sensitivity despite achieving target refraction due to irregular astigmatism and surface disturbance;
- A prolonged stabilization period (potentially 6–12 weeks) before achieving final refractive outcomes.

Clinical Implications:

1. Screen all keratoconus patients for dry eye disease before cataract surgery.
2. Consider preoperative treatment (preservative-free drops, punctal plugs, topical cyclosporine, or lifitegrast) for 2–4 weeks before biometry.
3. Obtain multiple biometric measurements, with careful attention to repeatability.
4. Accept wider refractive outcome targets (±0.75 D rather than ±0.50 D) in patients with moderate-to-severe DED.
5. Plan postoperative dry-eye management (aggressive lubrication and anti-inflammatory therapy) as integral to achieving optimal refractive outcomes.

We acknowledge that DED represents an important confounding factor in keratoconus biometry, and its impact on both measurement accuracy and refractive stability warrants increased clinical recognition and further research.

#### 3.6.2. IOL Haptic Design and Postoperative Anterior Chamber Depth

An important consideration in keratoconus IOL calculations involves the impact of IOL haptic design on postoperative anterior chamber depth (ACD) and effective lens position. Different haptic designs and materials may position the IOL differently relative to the cornea and ciliary body, potentially affecting refractive outcomes.

The altered geometry of keratoconic eyes, including deeper preoperative ACD and unusual corneal–lens spatial relationships, may interact with haptic design in unpredictable ways:

1. Haptic Flexibility: More flexible haptics may position differently in the accommodative system, particularly in younger keratoconus patients where ciliary body function persists.
2. Haptic Length: Shorter or longer haptics may achieve different postoperative ACD positions depending on the dimensions of the capsular bag.
3. Haptic Material: Different materials (PMMA, acrylic, or silicone) may have varying positioning stability in keratoconic capsular bags.
4. Capsular Bag Behavior: Keratoconic eyes may have altered capsule properties that affect IOL haptic positioning stability over time.

While most included studies utilized similar IOL models or did not specifically report IOL design parameters, this represents a potential source of variation in outcomes that warrants attention in future studies. We recommend the following:

- Reporting of specific IOL models and haptic designs in future keratoconus IOL studies;
- Investigation of whether particular haptic designs perform more consistently in keratoconic eyes;
- Consideration of standardized IOL choices when possible to reduce outcome variability;
- Recognition of haptic design as a potential confounder when interpreting formula performance comparisons.

Future prospective studies should specifically evaluate the interaction between IOL design and keratoconus geometry to optimize outcomes.

#### 3.6.3. Post-Keratoplasty Eyes

IOL power calculation becomes even more challenging after penetrating or lamellar keratoplasty. Krysik et al. evaluated 42 post-PK eyes in keratoconus and found that neither ultrasound nor optical biometry achieved the target refraction, with no statistically significant difference between methods [16]. The irregular graft–host junction, variable wound healing, and unpredictably adequate refractive power make these eyes particularly difficult to calculate for IOLs. In such cases, some authors recommend targeting slight myopia and counseling patients about the likelihood of residual refractive error requiring spectacle or contact lens correction.

#### 3.6.4. Eyes with Intracorneal Ring Segments

Patients with previously implanted intracorneal ring segments (ICRSs) for keratoconus management present unique biometric challenges. The ICRS alters corneal curvature, potentially affecting the accuracy of keratometry measurements. Bamdad et al. [30] compared IOLMaster 700 and Pentacam AXL in keratoconus eyes with ICRSs and found minor discrepancies in measurements between the devices [30]. When performing cataract surgery in eyes with ICRSs, consider (1) ensuring the ICRS position is stable before biometry, (2) obtaining measurements with multiple devices if possible, (3) using formulas that incorporate total keratometry, and (4) counseling patients about potentially increased refractive prediction variability.

#### 3.6.5. Fluctuations in Postoperative IOL Position

Recent evidence suggests that IOL position can fluctuate over time, even in stable pseudophakic eyes. Tutchenko et al. [70] (2024) documented significant fluctuations in anterior chamber depth and astigmatism in non-keratoconic pseudophakic eyes, with implications for long-term refractive stability and visual outcomes.

In keratoconus, where the capsular bag morphology is already altered and potentially less stable, such IOL position fluctuations could have magnified effects on refractive outcomes:

1. Altered Capsular Support: The keratoconic cornea’s unusual geometry and the potentially altered lens–zonular–ciliary body spatial relationships may predispose to greater IOL positioning variability.
2. Refractive Implications: Even small shifts in IOL position (0.1–0.2 mm) can produce meaningful refractive changes, which are particularly important in keratoconus, where prediction targets are already narrow (±0.25–0.50 D).
3. Astigmatism Changes: Fluctuating ACD could particularly affect toric IOL outcomes through rotational instability and a changing effective cylinder axis.
4. Long-term Outcomes: Most keratoconus IOL calculation studies report relatively short-term outcomes (1–3 months). Longer follow-up studies may reveal progressive refractive changes due to IOL positioning shifts.

#### 3.6.6. Clinical Implications for Keratoconus Patients

- Discuss potential for long-term refractive changes with patients preoperatively.
- Consider more conservative targeting strategies recognizing potential IOL position shifts.
- Perform refractive assessments at multiple time points (1 month, 3 months, 6 months, and 1 year) to characterize stability.
- In cases with progressive hyperopic shift over time, investigate potential IOL position changes with anterior-segment OCT.
- Recognize that toric IOL outcomes may evolve over time due to both IOL position shifts and capsular dynamics.

#### 3.6.7. Previous Corneal Collagen Cross-Linking

Corneal collagen cross-linking (CXL) has become the standard treatment for progressive keratoconus [56,57]. Theoretically, CXL could affect corneal refractive properties by altering stromal structure and possibly flattening the cornea slightly. However, most studies suggest that in stable, post-CXL eyes, standard biometry and IOL calculations can be performed successfully [23]. The key consideration is ensuring sufficient time has elapsed after CXL (typically 3–6 months) for corneal shape and refractive stability before proceeding with cataract surgery.

#### 3.6.8. Toric IOL Considerations

Keratoconus patients often have significant corneal astigmatism, raising questions about toric IOL use. Several studies have evaluated toric IOL outcomes in keratoconus [18,26,32,51]:

- Ton et al. [18] (2021): In 32 eyes with keratoconus receiving toric IOLs, visual acuity improved significantly, and subjective astigmatism decreased from −2.95 ± 2.10 D to −0.95 ± 0.80 D [18]. Barrett’s True-K formula with measured posterior corneal power resulted in 87.5% of eyes within ±0.50 D of the predicted refraction.
- Meta-analysis by Yahalomi et al. [32] (2022): A systematic review of toric IOL outcomes in keratoconus showed a substantial reduction in astigmatism and improvement in visual function [32]. However, postoperatively, the need for glasses or rigid contact lenses remained common due to residual irregular astigmatism.
- Fernandez-Munoz et al. [51] (2021): Long-term follow-up showed good refractive stability in mild and moderate keratoconus patients with toric IOLs, without evident progression signals [51].

Current consensus suggests toric IOLs can be considered in mild-to-moderate keratoconus with a stable, relatively regular astigmatic component confirmed by topography. Patients should be counseled that toric IOLs address the regular astigmatic component only and that irregular astigmatism may persist, potentially requiring continued spectacle or contact lens wear for optimal vision.

## 4. Discussion

This literature review comprehensively evaluates the challenges in biometry and IOL power calculation in keratoconus, synthesizing evidence from 20 studies encompassing 1596 eyes. The findings demonstrate that IOL power calculation in keratoconus represents a complex clinical problem stemming from multiple inter-related biometric measurement errors and violations of fundamental assumptions underlying standard IOL formulas.

### 4.1. Principal Findings and Clinical Implications

(a) Keratoconus-specific formulas provide superior accuracy: The most significant finding is the clear superiority of keratoconus-specific formulas, particularly Barrett’s True-K for keratoconus, over standard formulas. Across multiple independent validations, Barrett’s True-K consistently achieved median absolute errors of 0.10–0.35 D, compared to 0.47–0.90 D for non-keratoconus formulas [5,12,15]. This translates to clinically meaningful differences in refractive outcomes, with 49–72% of eyes within ±0.25 D using Barrett’s True-K versus 18–36% with standard modern formulas in all-comers analyses.
(b) The Kane keratoconus formula shows more variable performance across studies, with excellent results in Kane’s original series (MAE 0.81 D) but concerning myopic overcorrection in subsequent validations, particularly in moderate and severe cases [5,8,15]. This discrepancy may reflect differences in constant optimization, populations, or intrinsic formula characteristics, warranting further investigation and potential formula refinements.
(c) Barrett’s Universal II performs exceptionally well despite not being keratoconus-specific: A surprising finding is the excellent performance of Barrett’s Universal II, particularly in mild and moderate keratoconus. Helaly et al. [5] found that BUII achieved a MedAE of 0.34 D overall and even 0.46 D in severe cases‚ comparable to Barrett’s True-K KC. This likely reflects BUII’s sophisticated multivariable ELP prediction, which reduces over-reliance on corneal power. For surgeons without access to keratoconus-specific calculators, BUII represents an excellent alternative, particularly in mild-to-moderate disease.
(d) Total keratometry improves accuracy: The incorporation of total keratometry measurements significantly enhances IOL calculation accuracy by directly measuring posterior corneal power rather than relying on assumed ratios [2,13,25]. This advancement is particularly important in severe keratoconus, where the posterior corneal contribution becomes increasingly significant. The widespread adoption of swept-source OCT biometry with TK capability should be encouraged for keratoconus patients.
(e) Disease severity profoundly impacts calculation accuracy: A clear threshold effect exists, with reasonable accuracy achievable in mild keratoconus using multiple formulas but marked deterioration in severe disease. In cases with K > 55 D, only keratoconus-specific formulas and Barrett’s Universal II maintain acceptable performance [5,9,15]. This severity-dependent effect must inform preoperative counseling and surgical planning.
(f) Biometric measurement reliability decreases with severity: The systematic degradation of keratometry repeatability as disease progresses (particularly K > 55 D) represents a fundamental challenge that no formula can completely overcome [19,21,66]. Multiple repeated measurements, the use of different devices when possible, and appropriate patient counseling about increased prediction uncertainty are essential in advanced cases.

### 4.2. Comparison with Existing Literature

Our findings align with and extend recent reviews on this topic. Gershoni et al. [24] (2025) highlighted the importance of keratoconus-specific formulas and the challenges of altered corneal ratios. Singh et al. [11] (2023) emphasized that the Kane keratoconus formula performed better across all disease stages in their review. However, our analysis reveals more nuanced performance, with severity-dependent myopic overcorrection in some validations.

Nicholson et al. [23] (2024) provided comprehensive management recommendations, including preoperative corneal stabilization strategies. Our review complements this by providing detailed quantitative performance data stratified by severity to guide formula selection.

The network meta-analysis by Tian et al. (2025) provides high-level evidence supporting Barrett True-K formulas as optimal choices, consistent with individual study findings we analyzed [12] (2025).

### 4.3. Recommended Clinical Approach

Based on the reviewed evidence, we propose the following approach for IOL power calculation in keratoconus (also described in Supplementary Figure S1):

### 4.4. Preoperative Assessment

1. Confirm keratoconus diagnosis with corneal tomography (Pentacam or equivalent).
2. Document disease severity using a standardized classification.
3. Assess disease stability (no progression for ≥1 year).
4. Consider corneal cross-linking if progressive before proceeding with cataract surgery.
5. If intracorneal ring segments are present, ensure stable positioning.

### 4.5. Biometry

1. Use swept-source OCT biometry with total keratometry capability when available (IOLMaster 700, Anterion, or ARGOS).
2. Obtain multiple measurements (≥3 scans) and verify consistency.
3. Measure keratometry with Scheimpflug tomography (Pentacam, Oculus, Germany) for comparison.
4. Document anterior chamber depth, central corneal thickness, and white-to-white diameter.
5. In severe keratoconus (K > 55 D), recognize inherent measurement limitations and inform patients.

### 4.6. Formula Selection

First choice: Barrett’s True-K for keratoconus (with predicted or measured posterior corneal astigmatism, preferably using total keratometry input).

Second choice: Barrett’s Universal II (particularly if a keratoconus-specific formula is unavailable).

Third choice: Kane’s formula in mild cases or EVO 2.0 with TK in moderate cases.

Consider: SRK/T for comparison, especially in moderate-to-severe cases, where it has historically performed well

### 4.7. Targeting Strategy

- Mild KC (K ≤ 48 D): Target emmetropia to −0.25 D;
- Moderate KC (K 48–53 D): Target −0.25 to −0.50 D;
- Severe KC (K > 53 D): Target −0.50 to −0.75 D and counsel about increased prediction uncertainty.

### 4.8. IOL Selection

- Monofocal IOLs remain the first choice due to irregular optics.
- Toric IOLs can be considered in mild-to-moderate KC with a stable, relatively regular astigmatic component, recognizing that residual irregular astigmatism may persist.
- Premium multifocal/EDOF IOLs are generally contraindicated due to irregular corneal optics.

### 4.9. Patient Counseling

- Explain increased refractive prediction uncertainty compared to normal eyes.
- Discuss the likelihood of residual refractive error requiring spectacles or contact lenses.
- In severe cases, mention the possibility of IOL exchange if significant hyperopic surprise occurs.
- Emphasize that vision improvement is expected but that perfect uncorrected vision is less likely than in normal eyes.

### 4.10. Limitations

This systematic review has several important limitations that affect the strength and generalizability of the conclusions:

1. Predominance of Retrospective Studies (14 of 20): First, most of the included studies have retrospective designs. This introduces multiple sources of bias:
  - Selection bias in how patients were selected for cataract surgery;
  - Potential for selective outcome reporting;
  - Inability to control for confounding variables;
  - Unreliable documentation of baseline characteristics and measurement conditions.
2. Small Study Samples: Many included studies enrolled relatively small patient cohorts with inherent biases, particularly for severe keratoconus subgroups:
  - Limited statistical power for subset analyses;
  - Results susceptible to outlier influence;
  - Difficulty achieving representative patient populations;
  - Sample sizes ranging from 12 to 637 eyes, with most <100.
3. Publication Bias: Several sources of publication bias likely exist:
  - Studies demonstrating successful outcomes with newer formulas are preferentially published;
  - Null results or disappointing outcomes with established formulas less likely to be published;
  - Industry-sponsored studies potentially over-represent positive findings;
  - No systematic search of gray literature.
4. Heterogeneity Preventing Meta-Analysis: As discussed above, substantial methodological selection and data collection bias may be relevant. Second, heterogeneity precluded quantitative meta-analysis.
5. Inconsistent Constant Optimization: IOL formula constants were inconsistently optimized or reported to exist in keratoconus classification systems, with some studies using Amsler–Krumeich classification and others using K-based or tomography-based classifications, limiting direct comparability. Third, constant optimization for IOL formulas was inconsistently performed across studies, potentially affecting formula performance rankings.
6. Limited Data on Severe Keratoconus: Disproportionate representation of mild-to-moderate cases limits precision of recommendations for the most challenging cases.
7. Short Follow-up Periods (most included studies): Fourth, the variety of biometry devices used across studies introduces another source of heterogeneity. Fifth, follow-up durations varied, with some studies reporting relatively short-term 1-month outcomes (while other reported 3–6-months postoperative outcomes), limiting assessment of long-term though refractive stability.
8. Inconsistent Outcome Metrics: Variability in outcome reporting limits direct comparisons between studies.
9. Limited Diversity in Geographic and Ethnic Populations: Most studies being conducted in specific geographic regions limits generalizability.
10. Device-Specific Limitations: Different devices demonstrate that varying performance concerns are less pronounced in keratoconus.
11. Post-Refractive Surgery Eyes: Minimal specific data were reviewed on keratoconus patients with prior to post-refractive surgery.
12. Toric IOL Data: Limited specific data on toric IOL performance in keratoconus were reviewed.
13. Variable Treatment of Formula Versions: Some formulas exist in multiple versions and have been inconsistently tested.
14. Lack of Standardization: No consensus protocols are available for keratoconus biometry and IOL calculations.

Despite these limitations, the included studies provide the best available evidence on keratoconus IOL calculations, and our findings represent the most comprehensive synthesis available of this literature to date.

### 4.11. Novel Contributions of This Review

This 2025 review advances the field beyond previous publications through several key contributions:

1. Updated Evidence Base: This review incorporates 2023–2025 literature not covered in previous reviews. Sixth, most studies included predominantly mild-to-moderate cases, with relatively few severe keratoconus eyes, limiting statistical power for subgroup analyses by previous reviews, including pivotal recent studies and the Tian et al. [12] (2025) network meta-analysis.
2. Quantitative Severity Stratification: This review provides detailed severity-stratified quantitative outcomes, enabling evidence-based formula selection.
3. Total Keratometry Synthesis: This review includes a comprehensive synthesis of evolving total keratometry evidence, demonstrating consistent improvement in accuracy.
4. Kane Formula Re-evaluation: This work also characterizes complex Kane keratoconus formula performance and identifies myopic overcorrection in moderate and severe cases.
5. Barrett Universal II Surprise Finding: This review highlights the unexpectedly excellent performance of a non-keratoconus-specific formula.
6. Comprehensive Special Considerations: This review represents the first systematic synthesis of post-keratoplasty, ICRS, and post-CXL considerations, as well as ocular surface disease and IOL position stability effects.
7. Actionable Clinical Framework: This work provides a severity-stratified clinical algorithm for implementation.
8. Explicit Methodology and Limitations: This review also includes a transparent discussion of the review methodology and its limitations.
9. Future Research Agenda: Finally, this review presents detailed research priorities for advancement of the field.

Thus, this review represents the most current, evidence-based, and clinically applicable synthesis of keratoconus IOL calculations available.

### 4.12. Future Directions

Several essential research directions emerge from this review:

(a) Formula refinement: The variable performance of the Kane KC formula across studies suggests the need for either constant optimization strategies specific to keratoconus or algorithmic refinements to prevent myopic overcorrection in severe cases. Collaboration between formula developers and clinicians with large keratoconus databases could facilitate these improvements.
(b) Artificial intelligence approaches: Machine learning algorithms trained on large datasets of keratoconus IOL calculations might identify patterns and predictive factors not captured by current formulas. Early work with AI-based IOL formulas in post-refractive eyes shows promise [35,36], and similar approaches could benefit keratoconus calculations.
(c) Standardization of measurements: The development of consensus guidelines for biometry measurement protocols in keratoconus would improve study comparability and clinical standardization. This could include recommendations for the number of measurements, quality criteria, preferred devices by severity stage, and handling of discrepant measurements.
(d) Severity-adjusted modifications: Rather than single keratoconus-specific formulas, severity-stratified algorithms or formula weights might optimize accuracy across the disease spectrum. The current observation that different formulas perform optimally at different severity levels suggests benefit from adaptive approaches.
(e) Ray-tracing and wavefront-based calculations: Theoretical IOL calculations based on actual corneal topography data (ray tracing) rather than simplified K values might improve accuracy in irregular corneas. Several studies have explored this in post-refractive eyes, with promising results [53,71].
(f) Long-term outcomes: Most studies report 1–3-month refractive outcomes. Longer follow-up studies assessing refractive stability over 1–5 years would help determine whether IOL calculations remain accurate as patients age and as keratoconus potentially progresses (though most cataract patients have stable disease).
(g) Prospective trials: Well-designed prospective studies with standardized protocols, predefined formula comparisons, masked refractive examiners, and adequate sample sizes across severity strata would provide higher-level evidence than current retrospective series.

## 5. Conclusions

Intraocular lens power calculation in keratoconus poses substantial challenges due to altered corneal geometry, variable measurements, and violated formula assumptions. However, recent advances in both biometry technology and formula development have significantly improved outcomes. Keratoconus-specific formulas, particularly Barrett’s True-K for keratoconus, demonstrate superior accuracy, with median absolute errors of 0.10–0.35 D compared to 0.47–0.90 D for standard formulas. Barrett’s Universal II also performs exceptionally well despite not being keratoconus-specific. The use of swept-source OCT biometry with total keratometry capability enhances accuracy by incorporating posterior corneal measurements.

Disease severity profoundly impacts calculation accuracy, with marked performance deterioration in severe keratoconus (K > 55 D), necessitating careful patient counseling about increased refractive prediction uncertainty. A multimodal approach combining advanced biometry devices, keratoconus-specific formulas, comparison across multiple calculations, and conservative refractive targeting with comprehensive patient education optimizes outcomes in this challenging population. Surgeons should preferentially use Barrett’s True-K for keratoconus or Barrett’s Universal II, employ swept-source OCT biometry when available, and adjust expectations and targets based on disease severity.

## Figures and Tables

**Table 1:** List of included studies.

Study (First Author, Year)	Study Design	Number of Eyes/Studies	Mean Age (Years)	Biometry Device Used	Focus	Reason for Inclusion
Kane et al., 2020 [8]	Retrospective case series	147 eyes	Not mentioned	Scheimpflug + IOLMaster comparison	IOL formula accuracy in KC	KC-specific formula validation
Vandevenne et al., 2022 [15]	Multicenter retrospective study	57 eyes	68	Scheimpflug tomography (Pentacam)	Barrett True-K vs. Kane KC accuracy	Primary data on Barrett’s True-K performance
Heath et al., 2023 [2]	Retrospective cohort study	87 eyes	67.2	IOLMaster 700 (SS-OCT)	TK vs. standard K with newer formulas	Total keratometry evaluation
Helaly et al., 2025 [5]	Retrospective case series	99 eyes	55.4	IOLMaster 700 (SS-OCT) + Pentacam	KC severity effect on formula accuracy	Severity-stratified outcomes
Tian et al., 2025 [12]	Network meta-analysis	637 eyes (9 studies)	43.7–68 (range across 9 studies)		Network meta-analysis of IOL formulas	High-level evidence synthesis
Watson et al., 2014 [9]	Retrospective case series	92 eyes	63	Scheimpflug + Placido + IOLMaster	Cataract surgery outcomes in KC	Long-term outcome data
Savini et al., 2019 [17]	Retrospective study	57 eyes	66.5	Multiple devices	IOL formula comparison in KC	Formula comparison data
Ton et al., 2021 [18]	Retrospective case series	32 eyes	51.2	Scheimpflug tomography	Toric IOL calculation in KC	Toric IOL-specific outcomes
Yokogawa et al., 2024 [14]	Multicenter study	131 eyes	56.8	IOLMaster 700 (SS-OCT)	Multicenter formula validation	Large multicenter validation
Parra-Bernal et al., 2024 [13]	Retrospective study	55 eyes	52.1	IOLMaster 700 (SS-OCT) + Pentacam	Total vs. standard keratometry	TK vs. SK comparison
Ghiasian et al., 2019 [1]	Literature review	Review	NA (literature review)		IOL calculation review	Comprehensive review of challenges
Hashemi et al., 2015 [19]	Prospective case series	23 eyes	38.4	Pentacam, IOLMaster, Orbscan, Manual keratometer	Keratometry repeatability by severity	Biometry repeatability data
Singh et al., 2023 [11]	Comprehensive review	Review	NA (comprehensive review)		Surgical management update	Current practice guidelines
Kamiya et al., 2018 [20]	Multicenter retrospective study	64 eyes	54.3	Pentacam + IOLMaster	Multicenter predictability study	Corneal topography-based calculations
Gustafsson et al., 2020 [21]	Prospective cohort study	78 eyes	45.2	Pentacam HR	Measurement repeatability by severity	Measurement error quantification
Hashemi et al., 2020 [22]	Systematic review/meta-analysis	22 studies	N/A (systematic review/meta-analysis)		ACD difference meta-analysis	Biometric parameter analysis
Krysik et al., 2019 [16]	Retrospective comparative study	42 eyes	58.6	IOLMaster 500 + Ultrasound	US vs. optical biometry post-PK	Biometric device comparison
**Nicholson et al., 2024 [23]**	Comprehensive review	Review	N/A (comprehensive review)		Review of management concepts	Current management strategies
**Gershoni et al., 2025 [24]**	Review article	Review	N/A (Review article)		Recent advances in IOL calculations	Recent formula advances
**Chalkiadaki et al., 2022 [25]**	Agreement study	50 eyes	49.7	IOLMaster 700 + Pentacam AXL	IOLMaster 700 vs. Pentacam agreement	Device measurement agreement

IOL = intraocular lens; KC = keratoconus; TK = true keratometry; K = keratometry; ACD = anterior chamber depth; US = ultrasound; PK = penetrating keratoplasty; N/A = not applicable.

**Table 2:** List of excluded studies.

Study (First Author, Year)	Study Type	Primary Reason for Exclusion
Leccisotti, 2006 [10]	Case series	Small sample size (*n* < 10) and high IOL exchange rate
Nanavaty et al., 2012 [26]	Case series	Small sample size (*n* < 10)
Jurkunas et al., 2006 [27]	Case series	Not focused on IOL calculation outcomes
Hoffer, 1993 [28]	Formula development	Formula development paper, not KC-specific
Lanier et al., 1992 [29]	Descriptive study	No IOL calculation outcomes
Bamdad et al., 2025 [30]	Pilot study with ICRS	Insufficient KC eyes without ICRS data
Fernández-Vega-Cueto et al., 2017 [31]	Review of surgical options	Refractive correction focus, not IOL calculation
Yahalomi et al., 2022 [32]	Meta-analysis	Duplicate data source (overlap with included studies)
Akman et al., 2016 [33]	Device comparison	Normal eyes only, not KC-specific
Multack et al., 2025 [34]	RCT biometry comparison	Comparison of normal eyes, not KC outcomes
Lou et al., 2024 [35]	AI formula development	AI formula without KC-specific validation
Stopyra et al., 2025 [36]	AI formula validation	No specific KC cohort analyzed
Bagheri et al., 2021 [37]	Biomechanical modeling	Modeling study, no clinical outcomes
Alejandre et al., 2022 [38]	Ray-tracing study	Optical analysis focus, not IOL calculation
Northey et al., 2021 [39]	Treatment algorithm	Treatment algorithm focus and limited IOL data
Fairaq et al., 2021 [40]	ICL outcomes	ICL not IOL focus
Kanellopoulos et al., 2013 [41]	Classification study	Classification focus, not IOL outcomes
Naderan et al., 2015 [42]	Classification study	Classification focus, not IOL outcomes
Chan et al., 2017 [43]	Device comparison	Device comparison in KC and limited IOL data
Çınar et al., 2013 [44]	Biometry comparison	Small KC sample and ultrasound focus
Yagci et al., 2015 [45]	Biometry repeatability	Limited KC sample and repeatability focus only
Tunç et al., 2021 [46]	Tomography repeatability	Repeatability only, no IOL outcomes
Kreps et al., 2020 [47]	Pentacam repeatability	Repeatability only, no IOL outcomes
Niazi et al., 2024 [48]	Fundamentals review	General KC review, not IOL-specific
Said et al., 2022 [49]	Six-month outcomes	Follow-up < 6 months
Moshirfar et al., 2023 [50]	Post refractive surgery	Post-refractive surgery focus
Fernandez-Munoz et al., 2021 [51]	Toric IOL long-term outcomes	Long-term stability and limited baseline data
Tamaoki et al., 2015 [52]	Posterior KC	Posterior KC (rare variant), *n* = 10
Lu et al., 2021 [53]	Posterior cornea review	General posterior cornea review
Coutinho et al., 2024 [54]	SimK vs. TK astigmatism	Astigmatism comparison, not IOL outcomes
Camellin et al., 2024 [55]	TCP prediction	TCP prediction method, no IOL validation
Naranjo & Manche, 2024 [56]	CXL review	CXL review, not cataract surgery focus
Papachristoforou et al., 2025 [57]	CXL review	CXL treatment review
Wang et al., 2024 [58]	ACD with visual-axis ectasia	Subset analysis (bilateral ectasia only)
Ortiz-Toquero et al., 2023 [59]	Scheimpflug vs. AS-OCT	Progression detection focus
Ashena et al., 2022 [60]	CXL technique comparison	No biometry data
Bardan et al., 2020 [61]	Classifying keratoconus	No biometry data
Bardan et al., 2018 [62]	Review	CXL with surface ablation review and no biometry data
Nanavaty et al., 2024 [63]	Algorithm development	Web-based algorithm for diagnosis but no outcome data
Salman et al., 2025 [64]	Keratoconus detection	Combined utilization of different parameters but no outcome data from biometry
Shalchi et al., 2015 [65]	CXL technique comparison	Epithelium off vs. epithelium on CXL comparison but no biometry outcome data

IOL = intraocular lens; KC = keratoconus; SimK = simulated keratometry; TK = true keratometry; ICRS = intracorneal ring segments; ICL = implantable contact lens; TCP = total corneal power; CXL = corneal cross-linking; AS-OCT = anterior segment optical coherence tomography.

## Data Availability

No new data were created or analyzed in this study. Data sharing is not applicable to this article.

## Supplementary Materials

The following supporting information can be downloaded at: https://www.mdpi.com/article/10.3390/diagnostics15243121/s1, Figure S1: Flow chart of recommendations for biometry calculations in keratoconus.
