# Supplementary figures and images for: Challenges in Biometry and Intraocular Lens Power Calculations in Keratoconus: A Review
Source: Diagnostics (Basel). 2025 Dec 8;15(24):3121. doi: 10.3390/diagnostics15243121 (PMC12731628; doi:10.3390/diagnostics15243121)

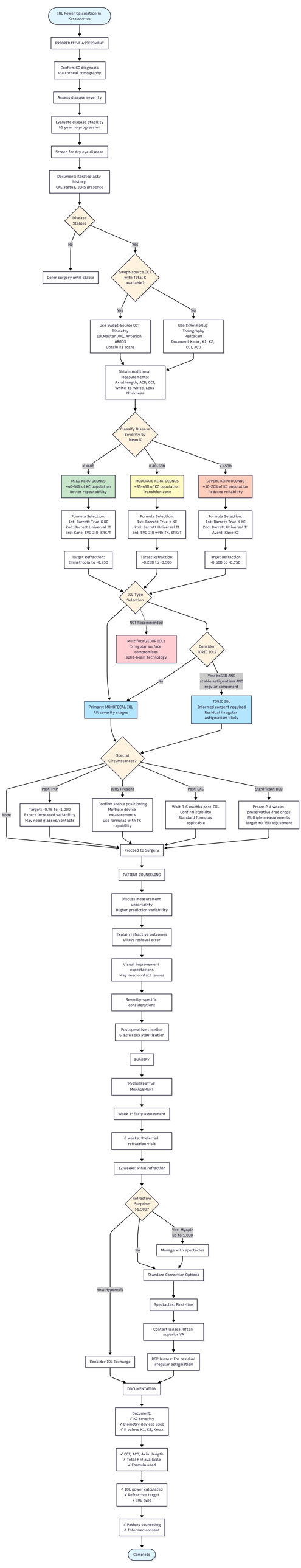

Supplement: Supplementary file 1 [file diagnostics-15-03121-s001.zip › diagnostics-3982475-supplementary.jpg]
